# Advances in Digital Physiotherapy and Rehabilitation: A Systematic Review

**DOI:** 10.7759/cureus.117639

**Published:** 2026-10-08

**Authors:** Sakshant Desai, Durga A Jagdale, Sandeep Shinde

**Affiliations:** 1 Department of Musculoskeletal Sciences, Krishna College of Physiotherapy, Krishna Vishwa Vidyapeeth (Deemed to Be University), Karad, IND

**Keywords:** artificial intelligence, digital health technology, digital rehabilitation, rehabilitation telerehabilitation, wearable technology

## Abstract

Digital health technologies have rapidly transformed physiotherapy rehabilitation by improving accessibility, patient engagement, remote monitoring, and continuity of care. Interventions such as telerehabilitation, wearable devices, artificial intelligence (AI), virtual reality (VR), smartphone-based applications, and remote monitoring systems are increasingly being integrated into musculoskeletal, neurological, and cardiovascular rehabilitation. This systematic review aimed to evaluate the clinical effectiveness of these digital health technologies by synthesizing evidence from randomized controlled trials (RCTs). A systematic search of PubMed/MEDLINE, Scopus, Web of Science, PEDro, Cochrane Library, and Google Scholar identified English-language RCTs published between January 2021 and July 2026. The review was conducted in accordance with the Preferred Reporting Items for Systematic Reviews and Meta-Analyses (PRISMA) 2020 guidelines, and the methodological quality of the included studies was assessed using the Cochrane Risk of Bias 2 (RoB 2) tool. Owing to heterogeneity in interventions, outcome measures, and study populations, findings were synthesized narratively.

Fourteen RCTs involving musculoskeletal, neurological, and cardiovascular populations met the eligibility criteria. Overall, digital health interventions significantly improved pain, physical function, mobility, range of motion, exercise adherence, functional independence, and quality of life. Telerehabilitation demonstrated outcomes comparable to conventional face-to-face physiotherapy for musculoskeletal disorders, wearable technologies enhanced motor recovery and functional performance in neurological rehabilitation, and remote cardiac rehabilitation improved exercise capacity, adherence, and health-related quality of life. Most included studies were judged to have some concerns about risk of bias, while one study demonstrated an overall low risk of bias. The findings suggest that digital health technologies are effective adjuncts or alternatives to conventional physiotherapy, enhancing accessibility, patient engagement, and rehabilitation outcomes. However, further high-quality multicenter RCTs with standardized intervention protocols and longer follow-up periods are required to strengthen the evidence base and support wider implementation in routine physiotherapy practice.

## Introduction and background

Digital health technologies have become increasingly integrated into contemporary physiotherapy rehabilitation, enabling remote assessment, individualized exercise prescription, continuous monitoring, and real-time communication between patients and clinicians. Digital physiotherapy or digital rehabilitation refers to the use of digital technologies, including telerehabilitation platforms, wearable devices, smartphone applications, artificial intelligence (AI), virtual reality (VR), biofeedback systems, and remote monitoring tools, to support the assessment, delivery, monitoring, and management of rehabilitation. These technologies can extend rehabilitation beyond traditional clinical settings and may improve access to physiotherapy for individuals with musculoskeletal, neurological, and cardiovascular conditions.

The use of digital rehabilitation has expanded in response to the increasing demand for accessible rehabilitation services and was further accelerated by the COVID-19 pandemic, which highlighted the potential of technology-assisted approaches to maintain continuity of care. Telerehabilitation is one of the most widely used forms of digital rehabilitation. Previous evidence suggests that exercise-based telerehabilitation can improve accessibility, convenience, and patient satisfaction, while producing clinical outcomes that may be comparable to those achieved through conventional face-to-face physiotherapy [1]. Telerehabilitation has also demonstrated potential cost-effectiveness in the management of musculoskeletal disorders [2].

Wearable technologies can continuously monitor physical activity, movement, gait, joint mobility, and exercise adherence, providing objective information that may assist clinicians in monitoring progress and modifying rehabilitation programs. Similarly, biofeedback-assisted rehabilitation can provide real-time information regarding movement and exercise performance and may support adherence and functional recovery [3,4].

Recent advances in AI have created additional opportunities for individualized rehabilitation. AI-assisted systems can support automated movement analysis, personalized exercise progression, and real-time performance feedback. Available evidence suggests that AI-assisted rehabilitation may contribute to reductions in pain and improvements in range of motion and functional performance in selected musculoskeletal populations; however, the magnitude and consistency of these effects may vary according to the technology, intervention, population, and outcome assessed [5]. This wording has been revised to avoid implying that AI-assisted rehabilitation uniformly produces the same effects across different clinical settings.

Digital health technologies have also been investigated across different physiotherapy specialties. Real-time telerehabilitation has demonstrated potential benefits in older adults with musculoskeletal conditions, while digital interventions and VR have been investigated for improving cognitive and motor outcomes following stroke [6-8]. Similarly, home-based cardiac telerehabilitation using remote monitoring and supervised digital programs has shown potential to improve exercise capacity, adherence, and health-related quality of life [9,10].

While wearables, VR, telerehabilitation, and AI have each been evaluated in previous systematic reviews, many of these reviews have focused primarily on individual technologies or specific patient populations. Consequently, evidence regarding the effectiveness of different digital health technologies across diverse physiotherapy specialties remains fragmented. A broader synthesis is therefore warranted to provide an integrated understanding of the therapeutic outcomes associated with these technologies across different rehabilitation populations. Thus, this systematic review aimed to evaluate the effectiveness of digital health technologies used as physiotherapy rehabilitation interventions, focusing on their effects on pain, physical function, mobility, and other patient-reported or performance-based outcomes in individuals with musculoskeletal, neurological, and cardiovascular conditions, based on evidence from randomized controlled trials (RCTs). Figure 1 illustrates the range of digital health tools currently used in physiotherapy rehabilitation.

**Figure 1:**
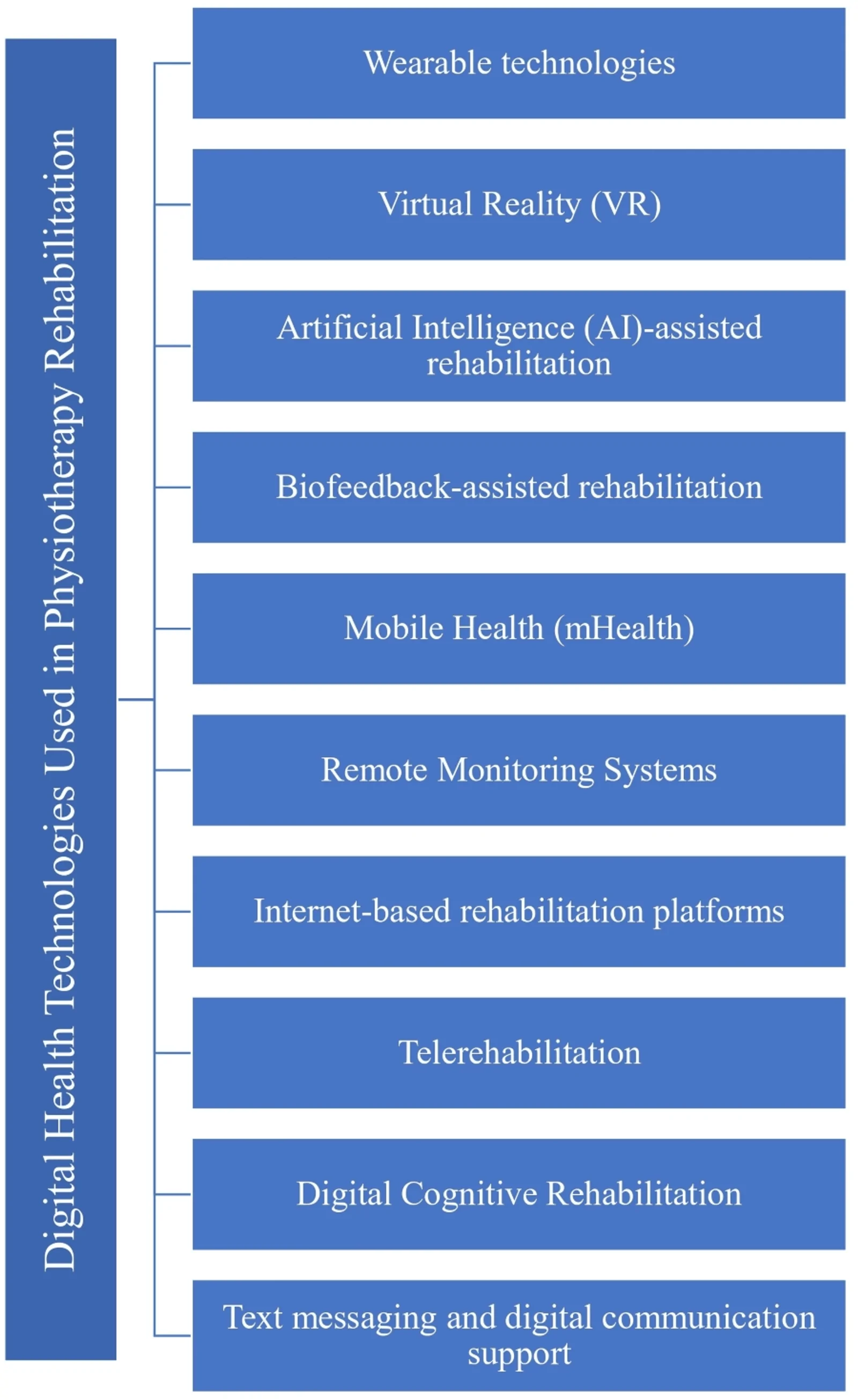
Digital health technologies used in physiotherapy rehabilitation The structure, design, and formatting of the figure were produced using Microsoft PowerPoint (Microsoft Corporation, Redmond, Washington, United States).

Therefore, by combining data from RCTs, this systematic review sought to assess the therapeutic efficacy of digital health technologies in physiotherapy rehabilitation. In order to provide a current body of evidence for physiotherapy practice, it evaluated their effects on pain, physical function, mobility, range of motion, exercise adherence, quality of life, and other clinically significant outcomes.

## Review

Methodology 

Study Design

This systematic review was conducted to evaluate the clinical effectiveness of digital health technologies in physiotherapy rehabilitation by synthesizing evidence from RCTs. The review protocol was prospectively registered with the International Prospective Register of Systematic Reviews (PROSPERO) (Registration No. CRD420261499567). The review was performed in accordance with the Preferred Reporting Items for Systematic Reviews and Meta-Analyses (PRISMA) 2020 guidelines [11]. Only peer-reviewed RCTs investigating digital health interventions, including telerehabilitation, wearable devices, AI, VR, and other technology-assisted rehabilitation approaches, were included. The methodological quality of the included studies was assessed using the Cochrane Risk of Bias 2 (RoB 2) tool, and the findings were synthesized using a narrative approach [12].

Search Strategy

A comprehensive electronic database search was conducted to identify relevant studies published between January 2021 and July 2026 using PubMed/MEDLINE, Scopus, Web of Science, PEDro, Cochrane Library, and Google Scholar. The search strategy was developed separately for each database using database-specific controlled vocabulary (e.g., MeSH terms) and free-text keywords related to digital health technologies and physiotherapy rehabilitation. The search terms included “digital health,” “digital rehabilitation,” “telerehabilitation,” “telephysiotherapy,” “artificial intelligence,” “AI-assisted rehabilitation,” “wearable devices,” “wearable sensors,” “smartwatch,” “mobile health,” “mHealth,” “smartphone application,” “virtual reality,” “biofeedback,” “remote monitoring,” “physiotherapy,” “physical therapy,” “rehabilitation,” “musculoskeletal disorders,” “stroke,” “neurological rehabilitation,” “cardiac rehabilitation,” “cardiovascular rehabilitation,” and “randomized controlled trial.” Boolean operators (AND/OR), field tags, truncation, and database-specific search syntax were applied as appropriate to maximize search sensitivity and specificity. The search strategy was adapted according to the indexing system and syntax requirements of each database. For Google Scholar, the first 100 results were screened by title and abstract. In addition, the reference lists of included studies and relevant systematic reviews were manually screened to identify potentially eligible studies. The complete electronic search strategies, database-specific search strings, filters, and exact search dates are presented in Table 1.

**Table 1:** Electronic database search strategy TS: topic search; TITLE-ABS-KEY: title, abstract, and keywords; PubMed: Public Medical Literature Analysis and Retrieval System; MEDLINE: Medical Literature Analysis and Retrieval System Online; PEDro: Physiotherapy Evidence Database

Database	Search strategy	Filters applied
PubMed/MEDLINE	("Digital Health" OR Telerehabilitation OR "Wearable Devices" OR "Artificial Intelligence" OR "Virtual Reality") AND (Physiotherapy OR Rehabilitation) AND ("Musculoskeletal Disorders" OR Stroke OR "Cardiac Rehabilitation") AND "Randomized Controlled Trial"	English, Humans, January 2021-June 2026
PEDro	digital health OR telerehabilitation OR wearable devices OR artificial intelligence AND physiotherapy rehabilitation	Clinical Trial, English
Scopus	TITLE-ABS-KEY ("Digital Health" AND Physiotherapy AND Rehabilitation AND (Musculoskeletal OR Neurological OR Cardiovascular))	English, January 2021-July 2026
Web of Science	TS=("Digital Health" AND Physiotherapy AND Rehabilitation)	English, January 2021-July 2026
Cochrane Library	("Digital Health" OR Telerehabilitation OR Wearable Technology) AND Rehabilitation	English, January 2021-July 2026
Google Scholar	"Digital health" physiotherapy rehabilitation wearable technology artificial intelligence randomized controlled trial	English, January 2021-July 2026

Eligibility Criteria

Inclusion criteria: Studies were included if they were RCTs involving participants undergoing physiotherapy rehabilitation for musculoskeletal, neurological, or cardiovascular conditions. Telerehabilitation, wearable technology, AI-assisted rehabilitation, VR, mobile health apps, biofeedback systems, and remote rehabilitation platforms were among the digital health technologies that were assessed in eligible studies. Studies had to report at least one clinically relevant outcome, such as pain, physical function, mobility, range of motion, disability, balance, motor recovery, exercise adherence, exercise capacity, or quality of life, and compare these interventions with standard rehabilitation, conventional physiotherapy, usual care, or another digital intervention. Only full-text publications written in English and published in peer-reviewed journals were included.

Exclusion criteria: Studies were excluded if they were systematic reviews, meta-analyses, narrative reviews, case reports, case series, conference abstracts, editorials, letters, study protocols, dissertations, or book chapters. Additionally, non-randomized, observational, cross-sectional, cohort, qualitative, and animal research were not included. Excluded were studies that failed to present clinically significant rehabilitation outcomes, lacked a comparison group, or did not assess digital health technologies in physiotherapy rehabilitation. Only publications written in English were taken into account. The most comprehensive and recent study was included when duplicate publications or overlapping datasets were found. Table 2 summarizes the inclusion and exclusion criteria as well as the eligibility requirements employed for study selection.

**Table 2:** Eligibility criteria of the included studies AI: artificial intelligence; VR: virtual reality; mHealth: mobile health; RCTs, randomized controlled trials

Component	Inclusion criteria	Exclusion criteria
Population	Human participants with musculoskeletal, neurological, or cardiovascular conditions requiring physiotherapy rehabilitation	Healthy participants, animal studies, or conditions not requiring physiotherapy rehabilitation
Intervention	Digital health technologies, including telerehabilitation, wearable device-based rehabilitation, AI, VR, mHealth, biofeedback-assisted rehabilitation, smartphone-based rehabilitation, and remote monitoring systems	Studies not involving digital health technologies or not related to physiotherapy rehabilitation
Comparison	Conventional physiotherapy, standard rehabilitation, usual care, or another digital rehabilitation intervention	Studies without a comparison group
Outcomes	At least one clinical outcome such as pain, physical function, mobility, range of motion, disability, balance, motor recovery, exercise adherence, exercise capacity, or quality of life	Studies not reporting clinical rehabilitation outcomes
Study design	RCTs, full-text articles, published in English between January 2021 and July 2026	Systematic reviews, meta-analyses, narrative reviews, case reports, case series, conference abstracts, editorials, letters, study protocols, dissertations, book chapters, observational studies, cohort studies, cross-sectional studies, qualitative studies, and non-English publications

Study Selection

Titles and abstracts were independently screened by two reviewers according to the predefined eligibility criteria. Articles considered potentially eligible were subsequently assessed independently by the same two reviewers at the full-text stage. Screening was performed independently and in duplicate, with reviewers blinded to each other’s decisions during the initial assessment. Any disagreements or uncertainties regarding eligibility were resolved through discussion and consensus between the two reviewers; when consensus could not be reached, a third reviewer was consulted. The reasons for exclusion at the full-text stage were recorded and documented.

Data Extraction

Data extraction was performed independently and in duplicate by two reviewers using a standardized, piloted data extraction form. The extracted data included author details, publication year, study design, sample size, population, interventions, outcome measures, and key findings. After independent extraction, the two reviewers compared their data for consistency. Any discrepancies or uncertainties were resolved through discussion and consensus, with consultation of a third reviewer when necessary.

Quality Assessment

The methodological quality and risk of bias of the included RCTs were assessed using RoB 2, the revised Cochrane risk-of-bias tool for randomized trials, developed by Sterne et al. (2019). The assessment covered five domains: bias arising from the randomization process, deviations from intended interventions, missing outcome data, measurement of the outcome, and selection of the reported result. Each domain was judged as low risk of bias, some concerns, or high risk of bias, and an overall risk-of-bias judgment was assigned according to the RoB 2 framework. The results of these assessments were considered during data synthesis and interpretation of the review findings.

Results

Due to substantial heterogeneity in study designs, sample sizes, participant populations, digital health technologies, comparators, outcome measures, and follow-up durations, a meta-analysis was not considered appropriate. Therefore, a structured narrative synthesis was performed. The included studies were first grouped according to clinical population (musculoskeletal, neurological, and cardiovascular conditions) and then categorized according to the type of digital health technology used, including telerehabilitation, wearable devices, mobile health applications, VR, AI, and biofeedback-based technologies. Within each technology and population category, studies were compared according to the intervention and comparator, outcome domains, follow-up duration, and direction of findings. Outcomes were synthesized descriptively by identifying patterns of benefit, no significant difference, or mixed findings across studies. Study characteristics, methodological quality, and risk of bias were considered when interpreting the consistency and strength of the findings. This approach allowed comparisons across clinically and technologically diverse interventions while avoiding inappropriate statistical pooling. Quantitative meta-analysis was not considered appropriate because pooling of data could have produced misleading estimates of treatment effects.

A total of 186 records were identified through database searching. After the removal of duplicate records and other ineligible records, 136 studies remained for screening. Following title and abstract screening, 64 full-text articles were assessed for eligibility. Of these, 50 studies were excluded due to inappropriate study design, an ineligible population, or failure to report relevant outcomes. Ultimately, 14 RCTs met the eligibility criteria and were included in the systematic review. The detailed process of study identification, screening, eligibility assessment, and inclusion, in accordance with the PRISMA guidelines, is shown in Figure 2.

**Figure 2:**
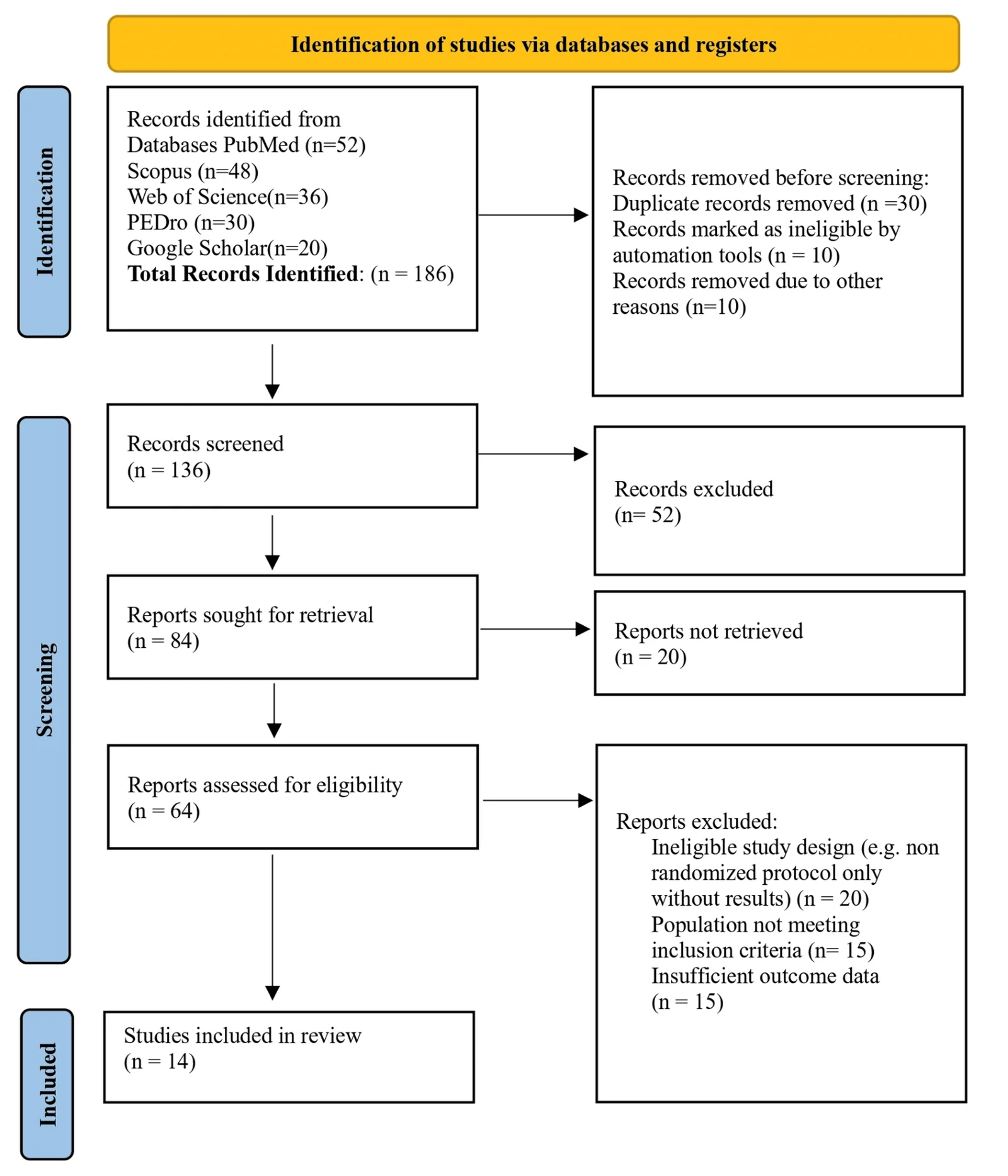
PRISMA flowchart PRISMA, Preferred Reporting Items for Systematic Reviews and Meta-Analyses

Fourteen RCTs evaluating digital health technologies in physiotherapy rehabilitation were included in this review. The therapies included wearable technology, smartphone-based apps, sensor-assisted therapy, telerehabilitation, AI-assisted rehabilitation, and cardiac telerehabilitation. Across musculoskeletal, neurological, and cardiovascular conditions, digital health interventions demonstrated potential benefits in selected outcomes, including pain, physical function, mobility, range of motion, exercise adherence, functional independence, and quality of life. However, the findings were heterogeneous across technologies, populations, and outcome measures, and several studies had some concerns regarding risk of bias. Therefore, the available evidence provides preliminary and supportive evidence that digital health technologies may serve as useful adjuncts to conventional physiotherapy, with their effectiveness varying according to the technology, clinical population, and rehabilitation context, as shown in Table 3.

**Table 3:** Overview of the 14 articles selected for this study AI: artificial intelligence; VAS: visual analog scale; SPADI: shoulder pain and disability index; ROM: range of motion; WOMAC: Western Ontario and McMaster Universities Osteoarthritis Index; TUG: timed up and go test; ODI: Oswestry Disability Index; OA: osteoarthritis; TKA: total knee arthroplasty; PROMs: patient-reported outcome measures; FMA: Fugl-Meyer assessment; BI: Barthel Index; BBS: Berg balance scale; FIM: functional independence measure; mRS: modified Rankin Scale; FAC: functional ambulation category; FMA-UE: Fugl-Meyer assessment-upper extremity; ARAT: action research arm test; MAL: motor activity log; BBT: box and block test; VO₂peak: peak oxygen uptake; 6MWT: six-minute walk test; CAD: coronary artery disease; CHD: coronary heart disease; NRS: numeric rating scale; KOOS-JR: knee injury and osteoarthritis outcome score-joint replacement; HADS: Hospital Anxiety and Depression Scale; TKS: Tegner Knee Score; FSS: fatigue severity scale; IPAQ-SF: International Physical Activity Questionnaire-short form; MDC: minimal detectable change; MCID: minimal clinically important difference; CI: confidence interval; MD: mean difference; NR: not reported; RCT: randomized controlled trial

Author (year)	Study design	Sample size	Population	Intervention	Duration/follow-up	Outcomes assessed	Main findings	Between-group difference/effect estimate	95% CI	P-value	MCID/clinical threshold
Tripuraneni KR et al. (2021) [13]	RCT	103	Patients undergoing primary total knee arthroplasty	Smartwatch with mobile application vs. standard postoperative rehabilitation	12 weeks	Knee ROM, PROMs, physical activity, patient satisfaction	Smartwatch-guided rehabilitation produced postoperative outcomes comparable to standard care while enhancing self-management and rehabilitation adherence	NR	NR	p=0.046 at 3 months; p=0.032 at 6 months; p=0.036 at 6 months	KOOS-JR MCID=8.02 points
Brouwers RW et al. (2021) [14]	RCT	300	Patients with coronary artery disease enrolled in cardiac rehabilitation	SmartCare-CAD cardiac telerehabilitation with relapse prevention vs. center-based cardiac rehabilitation	12 months	Physical activity, exercise capacity, cardiovascular risk profile, quality of life, long-term adherence	Both interventions improved physical activity, physical fitness, and quality of life; telerehabilitation produced outcomes comparable to centre-based rehabilitation and improved long-term adherence.	Between-group response: NR	NR	Within-group PAL p<0.01; between-group p=0.73	NR
Schwerz de Lucena D et al. (2022) [15]	RCT	74	Adults with chronic stroke and upper-limb impairment	Real-time wearable feedback to encourage affected-hand use vs. usual rehabilitation	6 weeks	MAL, accelerometer-measured arm activity, FMA-UE, adherence	Wearable feedback increased device-measured hand-use intensity and adherence, but clinical functional recovery was not clearly superior in the intervention group	Slope: 9.0 vs. 4.87 hand counts/hour/day	Regression CI reported graphically; exact CI NR	Feedback p=0.012; control p=0.059	MDC ≈31% of average daily hand-use intensity
Tore NG et al. (2023) [16]	RCT	60	Adults with knee osteoarthritis	Telerehabilitation exercise program vs. conventional physiotherapy	8 weeks	WOMAC, VAS, TUG, quality of life	Telerehabilitation improved pain, physical function, and quality of life, with outcomes comparable to face-to-face rehabilitation	Painkillers -4.62; 30-s chair stand +5.25; NRS pain -3.83; KOOS +27.66; HADS -5.33; TKS -4.79; FSS -1.91; IPAQ-SF +394.77	Painkillers -6.32 to -2.92; 30 CST 4.18 to 6.31; NRS -5.10 to -2.56; KOOS 15.44 to 39.88; HADS -8.20 to -2.46; TKS -8.21 to 1.36; FSS -2.62 to -1.20; IPAQ 19.05 to 770.48	Most p≤0.001; IPAQ p=0.040; TKS p=0.007	Outcome-specific MCIDs not consistently reported
Núñez-Cortés R et al. (2023) [17]	RCT	50	Adults with carpal tunnel syndrome	Telerehabilitation with pain neuroscience education vs. telerehabilitation alone	6 weeks	Pain, Boston Carpal Tunnel Questionnaire, hand function	Adding pain neuroscience education produced statistically significant and large effects on kinesiophobia and symptom severity; clinical importance remains uncertain because the MCID was not reported	ηp²=0.225 for kinesiophobia; ηp²=0.173 for symptom severity	NR	Kinesiophobia p=0.005; symptom severity p=0.013	MCID not reported
Villatoro-Luque FJ et al. (2023) [18]	Controlled Randomized Trial	68	Adults with chronic nonspecific low back pain	Telerehabilitation exercise program vs. center-based physiotherapy	8 weeks	Pain, disability, lumbar mobility, quality of life	Home-based telerehabilitation demonstrated outcomes comparable to clinic-based physiotherapy.	Exact between-group effect estimates NR	NR	Disability p=0.014; catastrophizing p<0.001; hip outcomes p=0.001-0.033	MCID not reported
Çelik EB (2024) [19]	RCT	60	Adults with subacromial pain syndrome	Manual therapy + exercise vs. synchronized telerehabilitation with self-manual therapy + exercise	6 weeks	VAS, SPADI, ROM, quality of life	Both interventions improved pain, function, and ROM; no significant difference was reported between manual therapy and telerehabilitation	NR	NR	NR in abstract	MCID not reported
Ho HJ et al. (2024) [20]	RCT	120	Patients with acute and subacute stroke	Wearable device-assisted rehabilitation + standard physiotherapy vs. conventional rehabilitation	12 weeks	FMA, mRS, BI, FAC, quality of life	Wearable-assisted rehabilitation showed statistically favorable improvements in motor recovery, gait, functional independence, and quality of life	Exact estimates NR	NR	Significant; exact values NR	MCID not clearly reported
Xiao C et al. (2025) [21]	RCT	120	Adults with chronic nonspecific low back pain	AI-based multimodal exercise telerehabilitation vs. conventional rehabilitation	12 weeks	Pain intensity, ODI, physical function, adherence	AI-assisted telerehabilitation reduced pain and disability and improved adherence; clinical importance should be interpreted against an established pain MCID	Most severe NRS week 4: adjusted MD -1.08; mean NRS -0.67; most severe NRS week 8: -0.95	-1.68 to -0.49; -1.19 to -0.15; -1.73 to -0.18	p<0.001; p=0.01; p=0.02	Pain MCID not explicitly established in reported abstract
Plavoukou T et al. (2025) [22]	RCT	72	Adults with knee osteoarthritis	Sensor-augmented telerehabilitation vs. conventional home exercise	8 weeks	WOMAC, muscle strength, balance, functional performance, psychosocial outcomes	Sensor-guided rehabilitation favored neuromuscular performance, functional ability, and psychosocial outcomes. Authors reported improvements exceeding clinically meaningful thresholds	Exact estimates NR	NR	Reported as significant/favorable; exact values NR	Authors reported improvements exceeding clinically meaningful thresholds
Lu Y et al. (2025) [23]	RCT	120	Patients with stroke undergoing rehabilitation	Wearable device-based intelligent stroke rehabilitation vs. conventional rehabilitation	12 weeks	FMA, BI, BBS, FIM, rehabilitation efficiency	Wearable intelligent rehabilitation showed statistically favorable improvements in motor function, balance, independence, and rehabilitation efficiency	Exact between-group estimates NR	NR	Significant; exact values NR	MCID NR
Toh FM et al. (2025) [24]	RCT	70	Adults with hemiparetic stroke	Wearable-based upper-limb rehabilitation with sensor feedback vs. conventional therapy	8 weeks	FMA-UE, ARAT, MAL, BBT, upper-limb activity	Wearable intervention improved upper-limb motor function and activity. Clinical importance remains uncertain without an established MCID	FMA-UE mean difference = 2.05 points favoring wearable intervention	NR	p=0.036; adherence p=0.038	MCID not reported
Nishio R et al. (2025) [25]	RCT	102	Patients with coronary artery disease participating in cardiac rehabilitation	Remote cardiac rehabilitation using wearable devices with or without weekly online coaching	6 weeks	VO₂peak, physical activity, exercise adherence, quality of life, satisfaction	Both groups improved exercise capacity, but weekly online coaching did not produce a significant between-group difference in peak VO₂ or anaerobic threshold	Peak VO₂: +1.6 vs. +0.6 mL/kg/min; AT VO₂: +1.0 vs. +1.3 mL/kg/min	NR	Between-group p=0.65 / p=0.90	MCID NR
Zhang S et al. (2025) [26]	RCT	120	Patients with coronary heart disease undergoing home-based cardiac rehabilitation	Smartwatch-assisted home-based cardiac rehabilitation vs. standard home-based rehabilitation	6 weeks	Adherence, 6MWT, quality of life, exercise compliance, cardiovascular fitness	Smartwatch-assisted rehabilitation improved adherence and several clinical outcomes; clinical significance cannot be established from p-values alone because effect estimates and MCIDs were not reported	Exact between-group estimates NR	NR	Adherence p<0.01; peak VO₂ p<0.01; GAD-7 p<0.01; PHQ-9 p<0.01; selected SF-36 p<0.05	MCID NR

Assessment of Risk of Bias

To ensure the reliability of the synthesized evidence, the risk of bias of all included studies was assessed using the Revised Cochrane Risk of Bias Tool (RoB 2). Each study was evaluated across five domains: the randomization process, deviations from intended interventions, missing outcome data, measurement of the outcome, and selection of the reported results.

The RoB 2 assessment indicated that the majority of the included studies were rated as having some concerns, primarily due to deviations from the intended interventions and the selection of reported results. The domains related to missing outcome data and outcome measurement were predominantly judged as low risk of bias across all studies. Only one study demonstrated an overall low risk of bias across all assessed domains. Overall, the included RCTs demonstrated some concerns regarding risk of bias, as shown in Table 4.

**Table 4:** Risk of bias assessment of RCTs using the Cochrane Collaboration’s RoB 2 tool RoB 2: risk of bias 2; RCTs, randomized controlled trials

Study	Randomization process	Deviations from intended interventions	Missing outcome data	Measurement of outcome	Selection of reported results	Overall RoB 2
Tripuraneni KR et al., 2021 [13]	Low risk	Some concerns	Low risk	Low risk	Some concerns	Some concerns
Brouwers RW et al., 2021 [14]	Low risk	Some concerns	Low risk	Low risk	Low risk	Some concerns
Schwerz de Lucena D et al., 2022 [15]	Low risk	Some concerns	Low risk	Low risk	Some concerns	Some concerns
Tore NG et al., 2023 [16]	Some concerns	Some concerns	Low risk	Some concerns	Some concerns	Some concerns
Núñez-Cortés R et al., 2023 [17]	Low risk	Some concerns	Low risk	Low risk	Low risk	Some concerns
Villatoro-Luque FJ et al., 2023 [18]	Some concerns	Some concerns	Low risk	Some concerns	Some concerns	Some concerns
Çelik EB et al., 2024 [19]	Low risk	Some concerns	Low risk	Some concerns	Some concerns	Some concerns
Ho HJ et al., 2024 [20]	Low risk	Some concerns	Low risk	Low risk	Some concerns	Some concerns
Xiao C et al., 2025 [21]	Low risk	Low risk	Low risk	Low risk	Low risk	Low risk
Plavoukou T et al., 2025 [22]	Low risk	Some concerns	Low risk	Low risk	Some concerns	Some concerns
Lu Y et al., 2025 [23]	Low risk	Some concerns	Low risk	Low risk	Some concerns	Some concerns
Toh FM et al., 2025 [24]	Low risk	Some concerns	Low risk	Low risk	Low risk	Some concerns
Nishio R et al., 2025 [25]	Low risk	Some concerns	Low risk	Low risk	Low risk	Some concerns
Zhang S et al., 2025 [26]	Low risk	Some concerns	Low risk	Low risk	Some concerns	Some concerns

Discussion

Digital health technologies have rapidly transformed physiotherapy rehabilitation by improving accessibility, patient engagement, treatment adherence, and clinical outcomes. This comprehensive analysis summarized the data from 14 RCTs evaluating digital health interventions for neurological, musculoskeletal, and cardiovascular rehabilitation. Overall, the findings varied according to the type of digital health technology and clinical population. Telerehabilitation demonstrated potential benefits for pain and physical function in musculoskeletal conditions, while wearable and sensor-assisted technologies showed improvements in mobility, motor function, and exercise adherence, particularly among individuals with stroke and other neurological conditions. AI-based and smartphone-supported interventions also showed promising effects on selected functional and pain-related outcomes. In cardiovascular rehabilitation, wearable and telerehabilitation approaches were associated with improvements in exercise capacity, adherence, and selected quality-of-life outcomes. However, the findings were heterogeneous across technology classes and populations, and the available evidence does not support attributing a uniform effect to all digital rehabilitation modalities. These results are in line with earlier systematic reviews and meta-analyses that have shown that digital rehabilitation increases access to rehabilitation services while producing clinical outcomes comparable to those of conventional in-person physical therapy.

Telerehabilitation in Musculoskeletal Rehabilitation

Telerehabilitation is a useful approach for managing postoperative rehabilitation after total knee arthroplasty, knee osteoarthritis, chronic nonspecific low back pain, carpal tunnel syndrome, and subacromial pain syndrome, according to evidence from musculoskeletal research. Tripuraneni KR et al. reported that smartwatch-guided therapy enhanced self-management and produced postoperative outcomes comparable to those of conventional rehabilitation [13]. Similarly, Tore NG et al. showed that telerehabilitation improved physical function and reduced pain in individuals with knee osteoarthritis [16]. Villatoro-Luque FJ et al. reported that home-based exercise delivered through telerehabilitation was as effective as center-based physiotherapy for chronic nonspecific low back pain, while Núñez-Cortés R et al. showed that adding pain neuroscience education to telerehabilitation further improved pain and hand function in individuals with carpal tunnel syndrome [17,18]. Çelik EB et al. found that synchronized telerehabilitation combined with conventional manual therapy resulted in similar improvements in shoulder function, range of motion, and pain in individuals with subacromial pain syndrome [19]. More recent research by Xiao C et al. showed that AI-assisted rehabilitation and therapist-supervised synchronous telerehabilitation improved pain, disability, treatment adherence, and patient satisfaction through customized exercise progression and real-time feedback [21]. Additionally, Plavoukou T et al. demonstrated that wearable sensors, motion trackers, and telemonitoring systems enhanced neuromuscular control, functional performance, exercise adherence, and quality of life [22]. Overall, these findings suggest that digital rehabilitation may serve as a dependable substitute or supplement to conventional physiotherapy for a variety of musculoskeletal conditions.

Digital Rehabilitation in Neurological Rehabilitation

Evidence from neurological research consistently supports the use of wearable technology in stroke recovery. Real-time wearable feedback enhanced daily use of the paretic upper limb and encouraged better adherence to rehabilitation, according to Schwerz de Lucena D et al. [15]. Building on these results, Ho HJ et al. discovered that patients with acute and subacute stroke had better motor function, gait, functional independence, and quality of life when they received wearable-assisted therapy [20]. Lu Y et al. found that an intelligent wearable rehabilitation system enhanced balance, activities of daily living, and rehabilitation efficiency, while Toh FM et al. showed improved upper-limb function and manual dexterity after sensor-based training [23,24]. By providing ongoing performance feedback and remote monitoring, wearable technology may facilitate intensive, task-oriented practice. These features could promote neuroplasticity, improve patient engagement, and facilitate rehabilitation outside traditional clinical settings.

Digital Health in Cardiac Rehabilitation

Digital health technologies have shown promise in improving home-based rehabilitation for individuals with coronary heart disease and coronary artery disease. According to Brouwers RW et al., cardiac telerehabilitation with relapse prevention produced outcomes comparable to those of typical center-based rehabilitation while also improving long-term adherence [14]. Similarly, wearable-based remote cardiac rehabilitation significantly increased exercise capacity, physical activity, and patient engagement, according to Nishio R et al. [25]. Positive outcomes were also observed with weekly online tutoring. In patients undergoing home-based cardiac rehabilitation, Zhang S et al. found that smartwatch-assisted rehabilitation enhanced exercise adherence, functional capacity, and health-related quality of life [26]. These results suggest that wearable monitoring, digital communication, and remote supervision can enhance long-term cardiovascular health, encourage self-management, and improve accessibility while facilitating successful cardiac rehabilitation.

Despite their potential benefits, digital rehabilitation interventions may be affected by socioeconomic barriers, limited device and broadband access, and differences in digital literacy, particularly among older adults. Disability-related accessibility needs, device costs, caregiver support, patient adherence, technology usability, and data privacy may also influence engagement. Therefore, these factors should be considered to promote equitable and patient-centered digital rehabilitation.

Overall, the included studies support the growing role of digital health technologies in physiotherapy rehabilitation across diverse clinical populations. These strategies improved access to rehabilitation services and showed positive effects on treatment adherence, functional recovery, and patient-reported outcomes. To determine long-term efficacy and maximize clinical application, however, further high-quality multicenter RCTs with standardized protocols and extended follow-up periods are needed.

Clinical Implications

The findings of this review indicate that digital health technologies are effective adjuncts to conventional physiotherapy across musculoskeletal, neurological, and cardiovascular rehabilitation. Features including remote monitoring, personalized exercise prescriptions, real-time feedback, and continuous therapist-patient communication enhance therapy accessibility, exercise adherence, and functional recovery while maintaining clinical efficacy. These technologies may be particularly beneficial for patients with limited mobility and individuals living in remote areas, as well as for healthcare systems seeking efficient and patient-centered rehabilitation programs. However, their implementation and potential benefits may depend on access to appropriate devices, digital literacy, reliable broadband connectivity, and patient preferences. To strengthen the body of evidence and promote wider clinical applicability, more rigorously designed multicenter RCTs with standardized intervention protocols, outcome measurements, and longer follow-up periods are needed.

Strengths

This systematic review provides a thorough assessment of digital health technologies in physiotherapy rehabilitation for cardiovascular, neurological, and musculoskeletal disorders. It provides high-quality evidence on the efficacy of wearable technology, smartphone-based applications, telerehabilitation, AI, and sensor-assisted rehabilitation because it exclusively includes RCTs. The transparency and dependability of the review findings were further improved by following PRISMA 2020 standards and evaluating methodological quality using the Cochrane Risk of Bias 2 (RoB 2) tool.

Limitations

Despite its strengths, this review has several limitations. Participant characteristics, rehabilitation methods, digital interventions, treatment duration, outcome measures, and follow-up periods varied significantly among the included studies, which hindered quantitative meta-analysis and precluded direct comparison of results. A narrative synthesis was therefore carried out. Because of problems with randomization and selective reporting, the majority of studies were classified as having some concerns in the RoB 2 assessment. Furthermore, linguistic bias may have been introduced by the inclusion of only English-language papers, and publication bias cannot be excluded because it was not formally assessed due to the absence of quantitative synthesis. Conclusions regarding long-term effectiveness are limited by the predominance of short- to medium-term follow-up among the included studies; therefore, the long-term efficacy of digital rehabilitation interventions remains uncertain. Lastly, variations in participant demographics and intervention strategies may limit the findings' applicability in other physiotherapy contexts.

Challenges

Physiotherapy is progressively incorporating digital health technologies, but there are still a number of obstacles to overcome. Direct comparison of study results is challenging due to contradictory evidence among rehabilitation specializations caused by differences in patient groups, intervention methods, outcome measures, and follow-up durations. The effectiveness and implementation of digital rehabilitation interventions may be influenced by the digital divide and socioeconomic barriers, including limited access to appropriate devices, device costs, inadequate broadband connectivity, and differences in digital literacy, particularly among older adults. Accessibility challenges for individuals with disabilities, the need for caregiver support, variations in patient adherence and technology usability, and concerns regarding data privacy and security may also affect engagement with these interventions. Successful implementation of digital rehabilitation should therefore consider patients’ socioeconomic circumstances, technological access and skills, accessibility requirements, caregiver support, privacy concerns, and individual preferences. To summarize the available data from RCTs and give physicians a better understanding of the clinical efficacy of digital health technologies in physiotherapy rehabilitation, a thorough systematic review is therefore required.

Future Directions

To strengthen the body of evidence, future research should prioritize large, multicenter RCTs evaluating technology-specific interventions using standardized core outcome measures and consistent intervention protocols. Studies should also include systematic reporting of adverse events, implementation outcomes, cost-effectiveness, patient experience and satisfaction, accessibility and digital equity, and longer-term adherence and clinical outcomes. The long-term efficacy, financial viability, and therapeutic use of wearable technology, AI, and remote monitoring systems require more research. The broader incorporation of digital health technology into standard physiotherapy practice will be facilitated by the creation of evidence-based therapeutic recommendations.

## Conclusions

Across the included RCTs, several digital rehabilitation interventions were associated with improvements in selected clinical and patient-reported outcomes across musculoskeletal, neurological, and cardiovascular rehabilitation. The findings of this review should be interpreted cautiously because of methodological and clinical heterogeneity across the included studies. The available evidence was limited by differences in digital rehabilitation technologies, intervention protocols, comparators, outcome measures, and follow-up periods. In addition, outcomes described as “comparable” should not be interpreted as demonstrating equivalence unless formal equivalence or non-inferiority testing was performed. Evidence specifically evaluating AI and VR was also limited relative to the broader range of technologies considered in this review. Furthermore, most included studies had some concerns regarding risk of bias. Thus, the findings should be considered supportive but preliminary, and stronger conclusions regarding the effectiveness of specific digital rehabilitation technologies require further well-designed research.
